# Transmission of hepatitis C virus among intravenous drug users in the Uppsala region of Sweden

**DOI:** 10.3402/iee.v4.22251

**Published:** 2014-01-15

**Authors:** Axel Danielsson, Navaneethan Palanisamy, Sultan Golbob, Hong Yin, Jonas Blomberg, Johan Hedlund, Staffan Sylvan, Johan Lennerstrand

**Affiliations:** 1 Section of Clinical Virology, Department of Medical Sciences, Uppsala University, Uppsala, Sweden; 2 Department of Communicable Disease Control and Prevention, Uppsala County Council, Uppsala, Sweden

**Keywords:** hepatitis C virus, transmission, intravenous drug users, phylogeny, NS5B, NS3

## Abstract

**Background:**

Epidemiology and transmission patterns of hepatitis C virus (HCV) are important subjects as we enter a new era of treatment with directly acting antivirals (DAAs). The highest prevalence of HCV in developed countries is found among intravenous drug users (IDUs), where unsafe needle sharing practices provide the main route of infection. Efforts to prohibit the continuous spread of HCV among these groups have been initiated by the community services and health care providers. Our goal was to understand how HCV was transmitted among IDUs within a limited population group. We provide a retrospective study (2005–2007) of the HCV transmission patterns in a population of IDUs in the Uppsala region of Sweden.

**Method:**

Eighty-two serum samples were collected from IDUs in Uppsala County. Our reverse transcription nested polymerase chain reaction (RT-nested PCR) and sequencing method enabled a comprehensive genetic analysis for a broad spectrum of genotypes of two relatively conserved regions, NS5B and NS3, that encodes for the viral polymerase and protease, respectively. HCV RNA in serum samples was amplified and sequenced with in-house primers. Sequence similarities between individuals and subgroups were analyzed with maximum likelihood (ML) phylogenetic trees. Published HCV reference sequences from other geographic regions and countries were also included for clarity.

**Results:**

Phylogenetic analysis was possible for 59 NS5B (72%) and 29 NS3 (35%) sequences from Uppsala patients. Additionally, we also included 15 NS3 sequences from Örebro patients, making a total of 44 NS3 sequences for the analysis. By analyzing the NS3 sequences, two transmission sets were found between the IDUs (>98% sequence identity), with one set consisting of two individuals and another set consisting of three individuals. In addition, the phylogenetic analysis done with our serum samples displayed clusters that distinguished them from the reference sequences.

**Conclusion:**

Our method seems to enable us to trace the HCV transmission between IDUs. Furthermore, the method is fairly independent of the time of infection because the method uses relatively conserved HCV sequence regions (i.e. NS5B and NS3).

More than 170 million people worldwide are infected with the hepatitis C virus (HCV) (1). The virus is primarily transmitted through blood and blood-related products, and HCV infection is most prevalent among intravenous drug users (IDUs) in developed countries. The mode of HCV transmission among IDUs varies; needles, pipes for snorting, rubber plugs, and cotton swabs are intermediates for infection (2, 3). HCV is the most common cause for liver transplantation in the United States (US Department of Health and Human Services). Several studies have investigated the transmission of HCV among groups of IDUs in order to correlate the social networks and the prevalence of HCV infections (4, 5). However, this has not been proven meaningful due to the continuous transmission among subsets of IDUs (5.

HCV is divided into seven (1–7) major genotypes with 30–40% sequence diversity (6) but with little morphological differences. All genotypes have a similar replication cycle in the host and are capable of causing asymptomatic and chronic infections. Subtypes within certain genotypes have 15–25% sequence diversity (6), although strains of the same subtype from different patients can display 2–10% sequence diversity. Even within a given patient, populations of HCV can display a genetic diversity of 3%. The high genetic diversity of HCV is due to (1) the error prone viral NS5B polymerase, (2) a high production rate of virions, and (3) intrapatient recombination (7). In general, tracing an outbreak (source of infection) or determining genetic relationships among HCV infected patients by phylogenetic analysis is difficult because of the high genetic diversity of HCV; the virus evolves independently in new hosts. To establish a genetic relationship among HCV sequences in different patients, the duration of infection in the patient has to be taken into account.

In order to amplify the genetic material for subsequent sequencing and phylogenetic analysis, it is necessary that the designed primers match the target region with enough specificity. Because the HCV genome is highly diverse, designing primers that match all the variants of the viral target sequence is a daunting task. When designing the amplification and sequencing primers, there has to be a sufficient balance between detection range (sensitivity) and specificity. This design often involves using degenerated primers with wobble positions (8). There are gene regions within the HCV genome that are more conserved than others. The 5′-untranslated region (5′-UTR) is one of the most highly conserved regions and is used in many commercial HCV nucleic acid detection assays (9, 10). The NS5B gene, encoding the viral polymerase, is also one of the regions within the HCV genome with the lowest sequence diversity. Mutations within this region may have a high impact on viral fitness because the replication cycle is dependent on the functionality of the polymerase. It has been found that the amino acid mutation rate within the NS5B region is 4.1*10^−4^ changes/site/year, compared to 1.4*10^−3^ changes/site/year for the entire HCV genome (11). The sequence of NS3 is also relatively conserved within the HCV genome because it encodes the essential viral protease.

The aim of our study was to investigate the transmission patterns of HCV among IDUs in the Uppsala region using phylogenetic analysis of the genes NS5B and NS3.

## Materials and methods

### Study population

In Sweden, on the local level, all newly diagnosed individuals with hepatitis C are reported by law to the medical officer (in this case, the co-author Staffan Sylvan) for the county, where the confirmatory diagnostic testing took place. On the national level, individuals are reported to the Swedish Institute for Control of Infectious Diseases (SMI).

Between the years 2005 and 2007, 82 serum samples were collected in Uppsala County from HCV-infected individuals that belonged to the IDU risk group. Written informed consent of the study population was not necessary because this study did not modify the existing diagnosis or the therapeutic strategy, and all serum samples were coded and could not be associated with an identifiable individual by the research team other than the county medical officer.

**Fig. 1:**
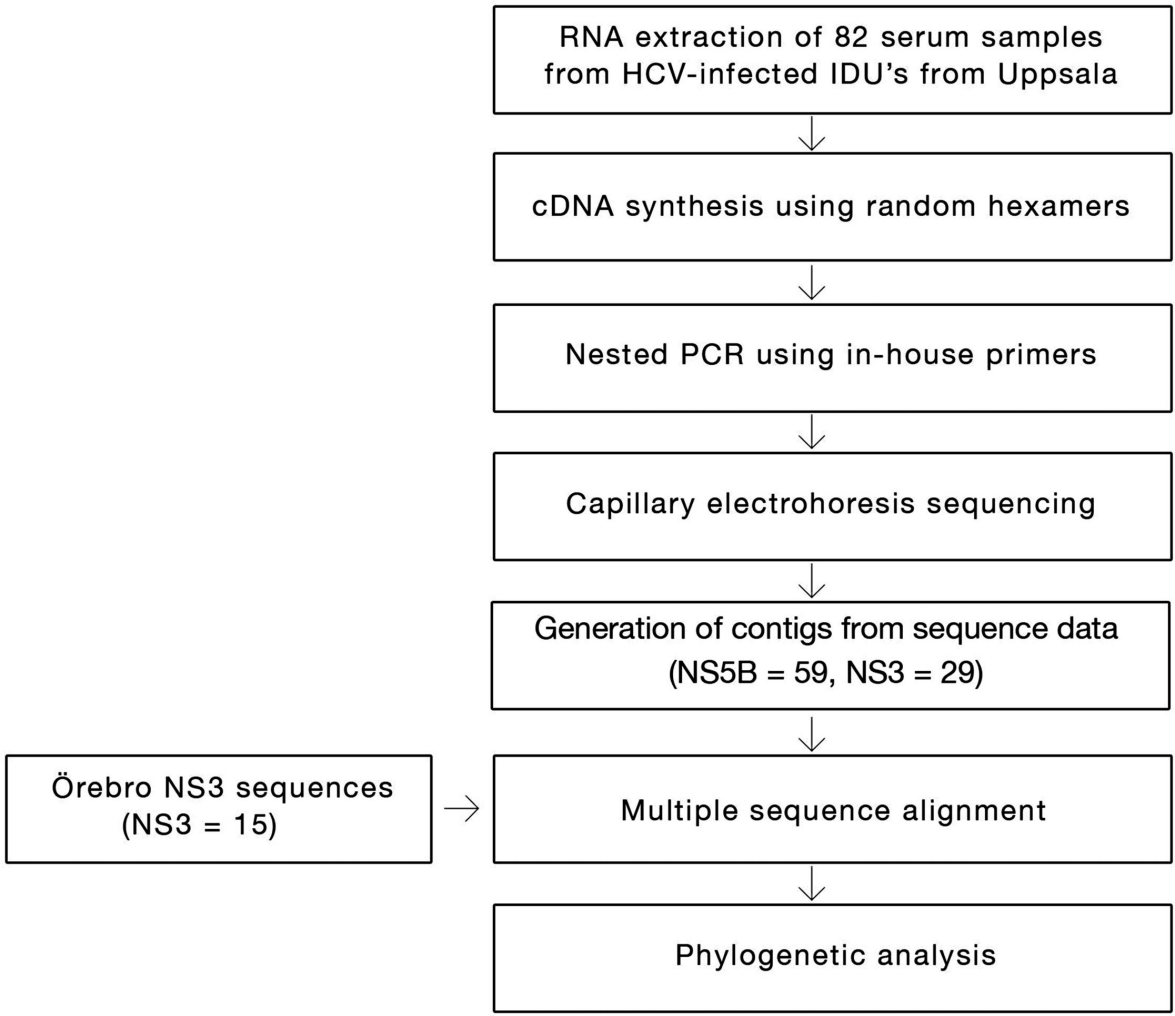
Flowchart overview of the methodology.

A cohort of 15 samples from Örebro University Hospital patients was added to the study to improve the phylogenetic analysis (see the flowchart, Fig. 1). They were collected for a separate Uppsala-Gävle-Örebro regional study to determine the prevalence of natural resistance in HCV to protease inhibitors in treatment naïve patients (12). Use of the Örebro samples was approved by the Regional Research Ethics Committee in Uppsala (Dnr 2009/023).

### RNA extraction and sequencing

RNA extraction from the serum samples (500 µL) was done using bioMérieux's NucliSENS^®^ easyMAG^®^ system according to the manufacturer's guidelines. The extracted RNA (20 µL) was stored at −70°C. We synthesized cDNA from RNA template (9 µL) with SuperScript^®^ III reverse transcriptase (Invitrogen) using random hexamers. RT (reverse transcription) consisted of pre-incubation at 25°C for 10 min, incubation at 42°C for 60 min, final incubation at 85°C for 5 min, and storage at 4°C. Nested-polymerase chain reaction (PCR) was performed with in-house primers targeting parts of the NS5B and NS3 regions using the TaqMan^®^ Universal PCR Master Mix (Applied Biosystems). Five μL of cDNA was used for the first round of nested-PCR. Concentration of primers was increased fourfold to compensate for the ambiguous positions. Thermocycling conditions for the first round of nested-PCR consisted of one cycle at 94°C for 4 min, followed by 35 cycles at 94°C for 30 s, 50°C for 30 s, 72°C for 1 min, a final extension cycle (72°C for 5 min), and a hold at 4°C. Two μL of first round PCR product was used for the second round of nested-PCR. Thermocycling conditions for the second round of nested-PCR consisted of one cycle at 94°C for 4 min, followed by 35 cycles at 94°C for 30 s, 55°C for 30 s, 72°C for 1 min, a final extension cycle (72°C for 5 min), and finally a hold at 4°C. The PCR products of second nested-PCR were detected by agarose gel (2%) electrophoresis. Positive samples were purified using QIAquick^®^ PCR Purification Kit (Qiagen) according to the manufacturer's guidelines. The purified products were sent to the Uppsala Genome Center for capillary electrophoresis sequencing (ABI 2700) using the nested primers (‘2nd Forward’ and ‘2nd Reverse’ in Table 1). BigDye^®^ Terminator v3.1 (Applied Biosystems) was used for the sequencing reactions. The reactions were carried out in 10 μL wells using 1 μL BigDye mix per reaction. Thermocycling conditions for the sequencing reactions consisted of one cycle at 94°C for 30 s, followed by 35 cycles at 94°C for 25 s, 50°C for 15 s, and 60°C for 2 min. All in-house primers of NS3 region for the first round and the second round nested-PCR are given in Table 1. The thermocycling conditions for first and second rounds of NS5B PCR have previously been described (13), and the degenerated primer sequences are given in Table 2.

**Table 1:** Sequences of the first and second round NS3 primers used

Primer	Sequence 5′ to 3′	Position in HCV genome
1st Forward	ATCACsTGGGGrGCrGAyAC	3,238–3,258 (in NS2 region)
1st Reverse	AAyTTGCCrTAkGTGGAGTAyGT	4,162–4,185 (in NS3 region)
2nd Forward	ACsGCrGCrTGygGGGACAT	3,257–3,276 (in NS2 region)
2nd Reverse	GTGCTCTTrCCGCTrCCrGT	3,983–4,004 (in NS3 region)

**Table 2:** Description of 1st and 2nd round NS5B primers used

Primer	Sequence 5′ to 3′	Position in HCV genome
1st Forward	CTACCATCA TGGCTAARAAYGAGGT	8,008–8,032 (in NS5B region)
1st Reverse	ATGATGTTATGAGCTCCARGTCRTA	8,673–8,697 (in NS5B region)
2nd Forward	TATGAYACCCGCTGYTTTGAC	8,256–8,276 (in NS5B region)
2nd Reverse	CCTGGTCATAGCCTCCGTGAA	8,616–8,636 (in NS5B region)

The NS5B and NS3 sequencing methods are currently used as routine for monitoring the HCV treatment, that is, genotype or subtype (NS5B) and resistance analysis (NS3) with new directly acting antivirals (DAAs) at the Department of Clinical Microbiology, Uppsala University Hospital.

### Phylogenetic analysis

Contig sequence (NS5B and NS3) for each isolate was obtained using DNA Baser Sequence Assembler V3.5.1 software (Heracle BioSoft 2010) and the result was converted into FASTA format. For each isolate, we obtained a minimum length of 365 nucleotides for the NS5B gene and 704 nucleotides for the NS3 gene. Jukes-Cantor (JC) was selected as the nucleotide substitution model using the ‘find model’ option in the MEGA 5.0 tool. Phylogenetic analysis was conducted using the same program (14). Bootstrap analyzes were performed with 1,000 replicates. For each genetic region, NS5B and NS3, phylogenetic trees were constructed using the maximum likelihood (ML) algorithm. Some samples could not be included in the analysis due to poor sequence quality. Reference sequences from geographically separated areas, corresponding to the 704 bp genetic region of NS3 analyzed in 44 genotype 1a/1b NS3 samples from Örebro and Uppsala, were retrieved from the Los Alamos HCV database and included in the analysis (Fig. 3).

## Results

### Genotype distribution

**Fig. 2:**
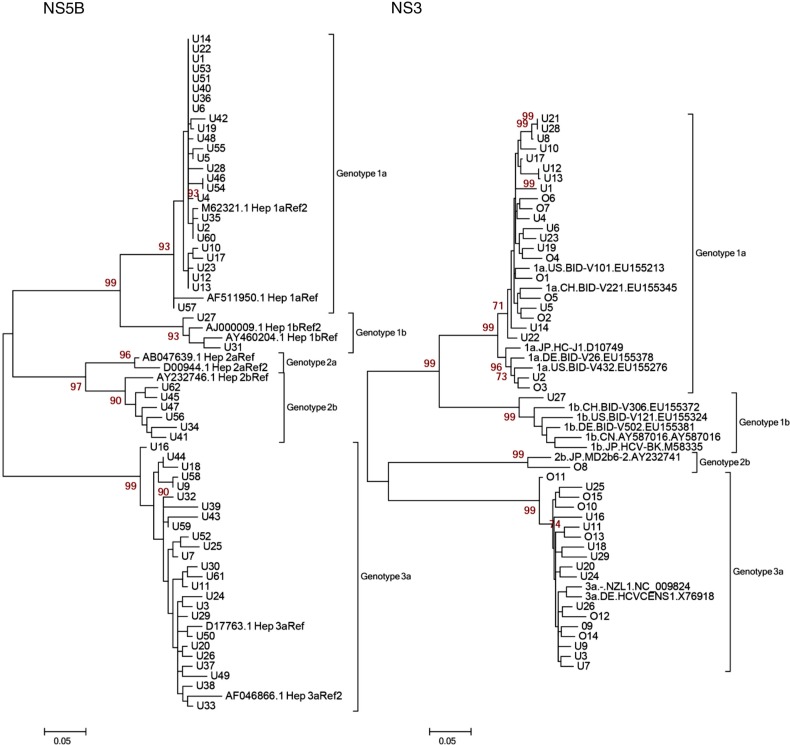
Maximum likelihood (ML) trees with 1,000 replicates. Genotype-reference sequences of the corresponding genetic region were retrieved from the Los Alamos HCV sequence database. Patient samples: U1-62 (Uppsala), O1-15 (Örebro). Left Panel: 59 NS5B patient sequences from Uppsala. Right Panel: 44 NS3 patient sequences from Uppsala and Örebro.

Phylogenetic analyses were possible using 59/82 (72%) NS5B sequences and 29/82 (35%) NS3 sequences from Uppsala County. To verify the findings in the NS5B analysis where we found a few suspected transmission sets, we analyzed a limited number of NS3 samples that we thought were of interest to see whether these transmissions could be found in this genetic region as well. Because the initial aim of our study was to use NS5B as the basis for the analysis, 39/82 samples (48%) were not included for the NS3 analysis. Also, due to poor sequence quality, 14 of 43 NS3 sequences (33%) were not included. The reason for poor sequence quality in 28% of the NS5B and 33% of the NS3 sequences might be due to the fact that the degenerated primers were not capable of detecting all the target regions and also to the use of random hexamers during the cDNA synthesis. To further strengthen this analysis, we included a few Örebro samples that had already been NS3 sequenced by us in a previous study (12). Fifteen NS3 sequences were added to the study from the Örebro hospital (total NS3 sequences for phylogenetic analysis = 44). Genotypes 1a, 2b, and 3a were the most common genotypes found in the cohort of 59 patients for the NS5B region (see Fig. 2, left panel). The patients were number coded with a U for Uppsala and an O for Örebro. Twenty-six patients (44%) had genotype 1a, two patients (3.4%) had genotype 1b, six patients (10%) had genotype 2b, and 25 patients (42%) had genotype 3a. In the NS3 cohort of 29 patients from Uppsala and 15 from Örebro, genotype distribution was similar: 23 patients (52.3%) had genotype 1a, one patient (2.3%) had genotype 1b, one patient (2.3%) had genotype 2b, and 19 patients (43.2%) had genotype 3a (Fig. 2, right panel).

### Nucleotide substitutions studied over time

NS5B and NS3 sequences from two patient samples taken at two different time points were aligned, and the number of identical sites was counted (Table 3). These two samples, U15 and U16, were first analyzed in 2006 and 2007, respectively, and were reanalyzed in 2009. NS5B and NS3 genes displayed 99.45 and 99.72% sequence identity, respectively, when the sequences were compared at two different time points in U16. One amino acid substitution had occurred in both NS3 sequences, although all other nucleotide changes were synonymous. In the NS5B sequences, all nucleotide changes were synonymous.

**Table 3:** Number of nucleotide changes in patient samples U15 and U16, analyzed 3 and 2 years apart in NS5B and NS3, respectively

Patient sample	No. of mutations in NS5B	No. of mutations in NS3	Time interval (in years)
U15	0/247	5/660	3
U16	2/365	2/704	2

### Defining transmission clusters and sets

**Fig. 3:**
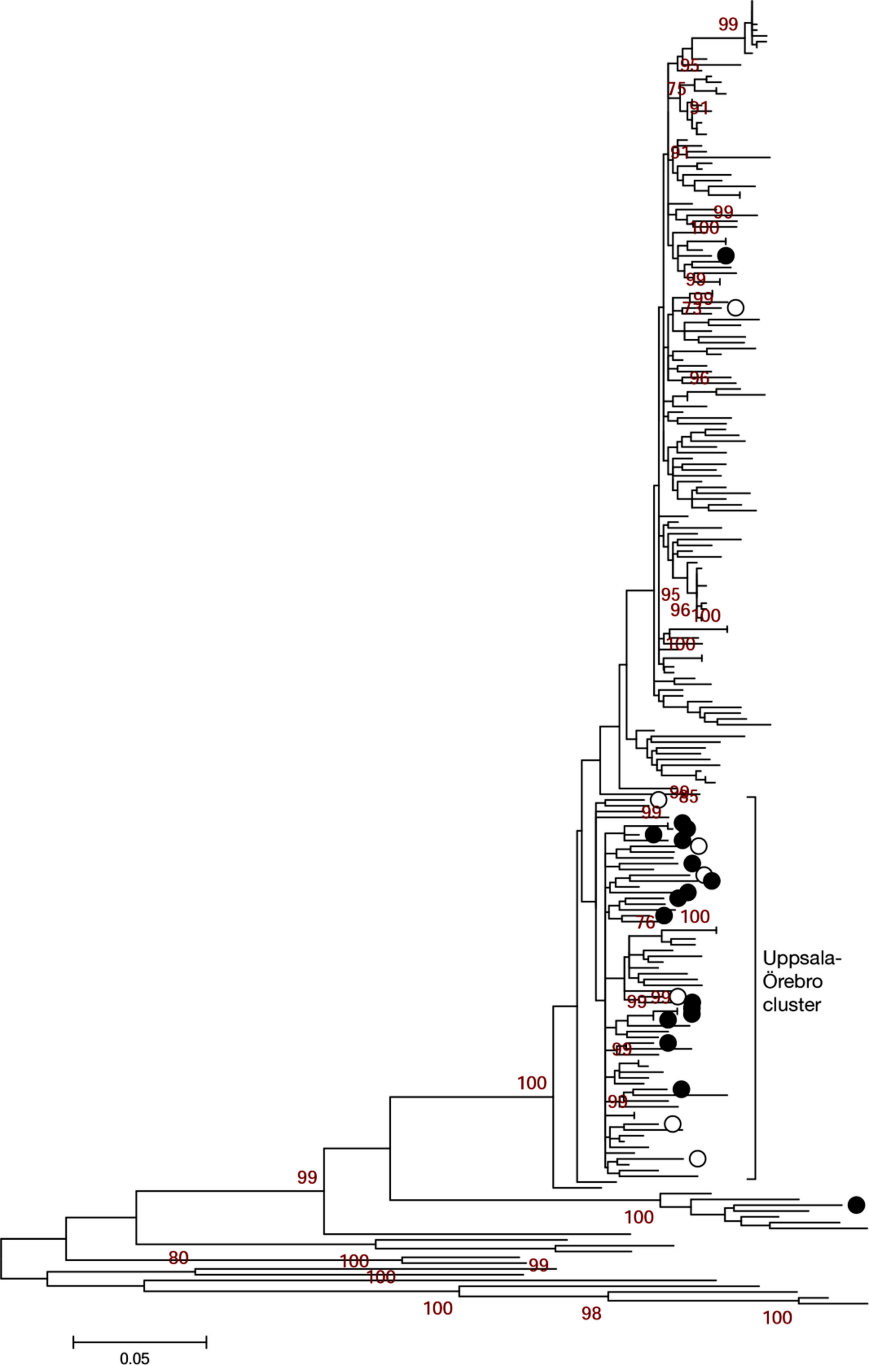
Uppsala-Örebro cluster. Maximum likelihood (ML) tree with genotype 1a/1b NS3 sequences displaying Uppsala patients (black rings; 16 genotype 1a and 1 genotype 1b) and Örebro patients (white rings; 7 genotype 1a) along with reference sequences from geographically separated areas. Only bootstrap-values above 60 are displayed. The Uppsala-Örebro cluster consisted of 15 Uppsala genotype 1a and 6 Örebro genotype 1a.

**Fig. 4:**
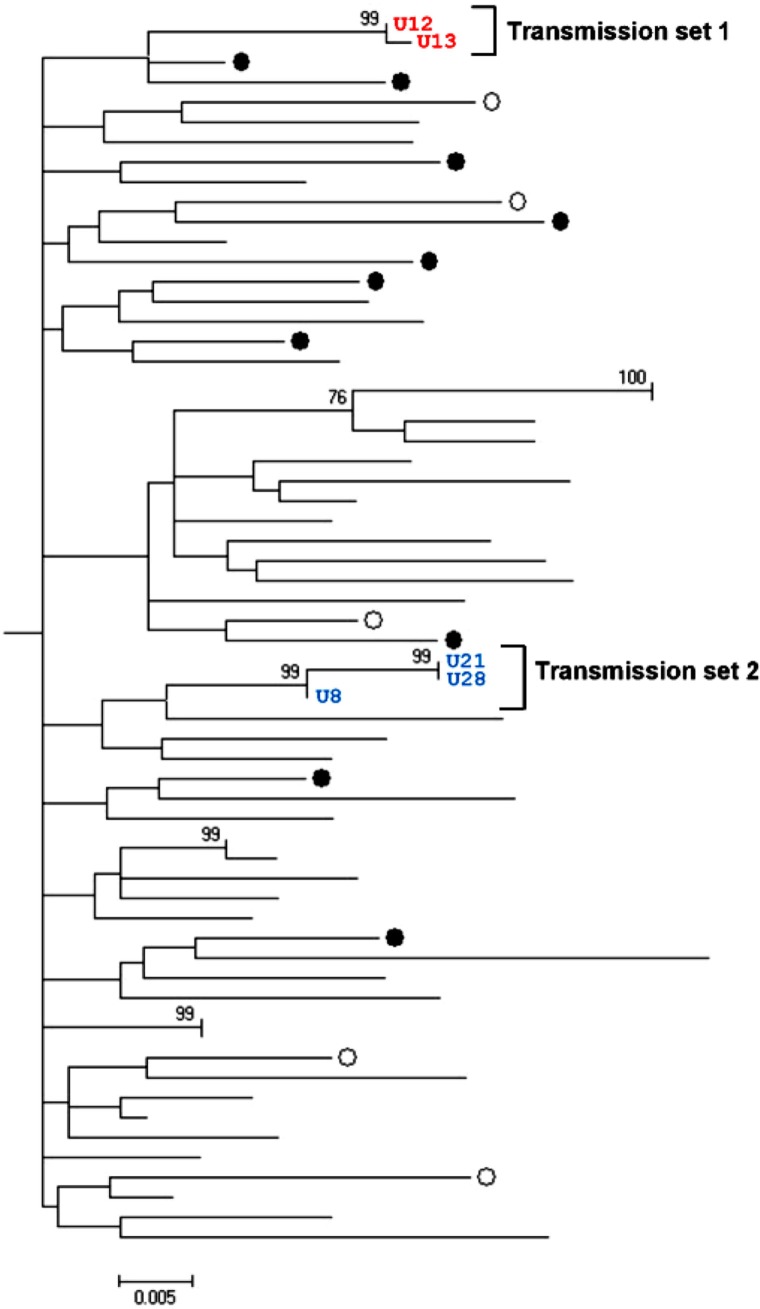
Transmissions set 1=red (U12 and U13) and 2=blue (U8, U21 and U28) within Uppsala-Örebro cluster share highly similar sequences within NS3 regions. Maximum likelihood (ML) tree with genotype 1a NS3 sequences displaying Uppsala patients (black rings or numbered) and Örebro patients (white rings). Only bootstrap-values above 60 are displayed.

A total of 515 NS3 genotype 1a/1b reference sequences were retrieved from the Los Alamos HCV database corresponding to the 704 bp region amplified in our experiment (15). In order to establish genetic correlations among the sequences in our study, the reference sequences were chosen for the geographical distance among them; our samples would make them unlikely to be related. The reference sequences originating from the United States, China, Japan, Denmark, Italy, Brazil, Cyprus, New Zealand, Indonesia, Egypt, Vietnam, South Africa, and Great Britain were used. These sequences were included in a ML tree (Fig. 3) along with our genotype 1a/1b cluster (Fig. 2, right panel) to distinguish clusters that potentially carried the same virus. One cluster contained 21 of the 44 NS3 sequences (47.7%) from Uppsala and Örebro. Within this cluster (Fig. 4), there were two sets of highly similar sequences from Uppsala (transmission sets 1=red, and 2=blue).

Transmission set 1 consisted of two individuals (U12 and U13) with 93.2 and 99.2% identical sites in the NS3 and NS5B region, respectively (data not shown). Transmission set 2 consisted of three individuals (U8, U21, and U28) with 98.2–99.7% and 96.3–98.8% identical sites in the NS3 and NS5B region, respectively (data not shown). Both transmission sets included patients who were diagnosed within a 3-year period. In transmission set 1, the patients (U12 and U13) were born the same year and diagnosed the same month. In transmission set 2, two of the patients (U21 and U28) were of the same age and were diagnosed during the same 10-month period. The third patient (U8) in the set was approximately 30 years older than the first two patients and was diagnosed 1 year before them.

## Discussion

Blood-borne viruses, such as hepatitis B and C (HBV, HCV), are common among illicit drug users and their sexual partners mainly because of the sharing of injection equipment and having unprotected sex with persons belonging to high-risk groups (16–18). Sero-epidemiological studies suggest that, in comparison with the general population, there is a high prevalence of chronic HCV infection among IDUs (19). The prevention of HCV infection is also a major public health concern because infected individuals are 75–85% more likely to develop chronic liver disease (20). We have previously shown that for hepatitis C, the sero-prevalence among IDUs was directly related to the number of years of injections (21). The role of the social network of IDUs in HCV transmission has been previously studied by another group (22). However, studies of the molecular epidemiology of HCV within local transmission networks of IDUs are scarce. Such studies are needed for a more comprehensive understanding of how HCV is transmitted.

In this study, the sequences generated from Uppsala patient sera showed a complete genotype correspondence for the phylogenetic trees between the two genetic regions NS5B and NS3 (Fig. 2). By comparing the sequence diversity in both regions, conclusions could be drawn about the difference in evolution rate between NS5B and NS3 and how HCV was likely to have been transmitted between individuals and subsets in the IDUs population. Reference sequences from separate epidemiological areas were included in the phylogenetic analysis to see whether a correlation between geographical and genetic distance could be established (Fig. 3). In a study of HIV infected IDUs in Sweden, it has been shown that most transmissions of HIV are local, with sporadic interactions among social networks from different parts of Sweden (23). Because the IDU population in Sweden is considered a mobile group, it is reasonable to believe that geographically separated populations of IDUs within Sweden are linked. To confirm this hypothesis, NS3 sequences from a cohort of 15 patients from Örebro University Hospital were included in the phylogenetic analysis of the NS3 region. An Uppsala-Örebro cluster is displayed in Fig. 3. However, we could not find any bootstrap support for an Uppsala-Örebro cluster. One should bear in mind that the geographically separated reference sequences (outside Sweden) used in the analysis from genotype 1a could be from clinical studies with NS3 protease inhibitors and could have affected the phylogenetic analysis due to occasional drug resistance mutations.

In Fig. 4, two transmission sets were identified. Of note, set 1 (U12 and U13) was also verified in the NS5B and NS3 panel in Fig. 2, whereas set 2 (U8/U21/U28) was only verified in NS3 (Fig. 2) because of the poor sequence quality of the U8 and U21 NS5B sequences. Patients within these sets displayed a high sequence identity in the two analyzed genetic regions. A set was considered epidemiologically connected if it was not split by any of the reference sequences from geographically separate areas. Two or more individual patients were considered a transmission set if the set in which they were grouped did not contain any of the reference sequences and the bootstrap value was greater than 90. Transmission set 2 contained two patients of approximately the same age and a third patient who was older. This pattern was anticipated because the experience from contact tracing is that younger IDUs (in this case, U21 and U28) often get their infection when sharing needles with older IDUs (in this case, U8) (24). Transmission set 1 (U12 and U13) displayed the same pattern as the two patients of the same age in transmission set 2; they were of the same age and were diagnosed during the same period of time. If there was an older IDU involved in this transmission, there is a possibility that he or she is included in this cohort of patients but was diagnosed earlier. If enough time has passed before this patient's sera was drawn, the sequence might have been altered to a point where not enough correlation can be established (e.g. less than 90% identical sites in the NS3 and NS5B region). When including reference sequences from geographically separated regions, distinct clusters formed with sequences from Uppsala and Örebro. However, it is difficult to know whether this implies that there was an ongoing transmission among IDUs in the Uppsala-Örebro region during 2005–2007 because the cluster lacks bootstrap support.

To estimate the nucleotide changes within a patient over time, serum samples were redrawn and analyzed from patients that previously had been diagnosed with HCV. Samples drawn on different occasions from each individual were compared to see how the NS5B and NS3 regions changed over a 2- to 3-year time span (Table 3). The comparison among sequences showed that NS5B and NS3 were conserved over time within patients (>99.4%). Although we only had access to two patient samples, the results indicated that the sequence region chosen was conserved for years in the individuals; therefore, the method could be considered reasonably independent of time of infection (in our study it was 2–3 years). However, in order to establish a more accurate substitution rate per year from this analysis, longer NS5B and NS3 fragments are required.

Conventional PCR has an intrinsic characteristic that complicates sequence analysis. When amplifying a chosen genetic region, the cDNA that is most prevalent in the test tube could be overrepresented in the resulting sequence. HCV is known to evolve into quasi-species within patients, and these subpopulations cannot be distinguished with conventional PCR (25). Considering this fact, it could be difficult to say whether a virion containing less prevalent RNA was transmitted from one IDU to another. If this was the case, the ‘recipient IDU’ will display a viral sequence that was not detectable in the ‘donor IDU’. However, capillary electrophoresis sequencing (resulting in consensus mixed sequences) should resolve this problem, unless the patient is double infected or has mixed HCV genotypes. An alternative method for such unusual double infection patients would be to use deep sequencing; for example, next generation sequencing has been used to study the transmission of HCV (26). This method, with its detection limit of less than 0.1% of quasi-species compared to capillary electrophoresis sequencing (20% of mixes), could be considered excessive for these types of studies. Thus, our capillary electrophoresis sequencing method should be sufficient for this study. Likewise, this type of method was already established to study the transmission of HIV-1 in IDUs (23).

In conclusion, our NS5B and NS3 sequencing and phylogenetic methods were combined to study the HCV transmission patterns in a population of IDUs in the Uppsala region. This method was shown to be fairly independent of the time of infection because the method uses relatively conserved HCV sequence regions but is still capable of discriminating sequences from different outbreaks. In the studied population, we have shown that two transmission sets could be detected, with sequences differing with only a few nucleotides. This indicates that these transmissions were interactions among social networks. These results suggest that it is possible to trace the source of transmission, which is of great importance because we now are entering a new era of HCV treatment. However, in order to improve the trace of transmission within IDU cohorts, further studies are needed involving a larger number of NS5B-NS3 sequenced samples sequencing longer regions and more updated medical record data.
